# Kaempferol-3-O-Rutinoside (K3R) Promotes Functional Rehabilitation by Modulating S100β and Inflammation Following Induced Traumatic Injury to Sciatic Nerve in Mouse Model

**DOI:** 10.3390/brainsci16101042

**Published:** 2026-09-29

**Authors:** Tehreem Iman, Humaira Muzaffar, Muhammad Zubair, Ghulam Hussain

**Affiliations:** 1 Neuroregeneration and Genetics Laboratory (NGL), Department of Physiology, Faculty of Life Sciences, Government College University, Faisalabad 38000, Pakistan; tehreemiman22@gcuf.edu.pk (T.I.); drhumairamuzaffar@gcuf.edu.pk (H.M.); 2 Health Biotechnology Laboratory, Department of Bioinformatics and Biotechnology, Faculty of Life Sciences, Government College University, Faisalabad 38000, Pakistan; muhammad.zubair1751@gmail.com

**Keywords:** Kaempferol-3-O-rutinoside, sciatic nerve injury, motor functions, sensory functions, oxidative stress, inflammation

## Abstract

**Highlights:**

**What are the main findings?**

- K3R improved sensorimotor functional recovery following sciatic nerve crush injury in mice.
- K3R reduced oxidative stress and proinflammatory cytokine levels and improved muscle fiber morphology after nerve injury.

**What are the implications of the main findings?**

- K3R shows promising neuroprotective potential by attenuating oxidative and inflammatory responses following peripheral nerve injury.
- K3R may represent a potential candidate for developing therapeutic strategies to enhance functional recovery after sciatic nerve injury.

**Abstract:**

Background: Peripheral nerve injury causes substantial functional impairment and represents a major health concern worldwide. Peripheral nerve injuries are sequelae of iatrogenic injuries, occupational trauma, and motor vehicle crashes. The available therapeutic approaches, including pharmacological treatment, surgical repair, and rehabilitation, are mostly management therapies rather than curative treatments. Consequently, functional recovery is never achieved. There is a dire need for new therapeutic approaches to facilitate functional restoration. Kaempferol-3-O-rutinoside (K3R) is a plant-derived flavonoid glycoside known for its potent anti-inflammatory and antioxidant properties. Flavonoids may promote tissue recovery following injury by modulating inflammatory responses and reducing oxidative stress. However, the effect of K3R on functional recovery following peripheral nerve injury has not yet been investigated. Therefore, this study aimed to evaluate the effects of K3R on early functional recovery following sciatic nerve crush injury in mice. Methods: Twenty-four BALB/c mice were equally allocated to three groups (*n* = 8): (1) Sham, (2) Ctrl (Control), and (3) K3R treatment group. K3R (2.5 mg/kg) was dissolved in DMSO and administered intraperitoneally once a day from the time of sciatic nerve injury until the conclusion of the experiment. The Ctrl and sham groups received an equal volume of DMSO according to the same dosing schedule. Sensorimotor function recovery was assessed using behavioral tests, including grip strength, pinprick, sciatic functional index (SFI), and hot plate tests. Hematological and biochemical assays were performed to assess total blood count and oxidative stress markers. Muscle fiber morphology was evaluated by morphometric analysis. Proinflammatory cytokines (TNF-α and IL-6) were measured as indicators of the systemic inflammatory response, whereas S100β protein levels were measured as a marker associated with neural injury and glial responses following sciatic nerve injury. Results: K3R treatment significantly enhanced early sensorimotor function recovery compared with the Ctrl group (*p* < 0.001). Significant differences in oxidative stress markers TAC (*p* < 0.001), TOS (*p* < 0.001), MDA (*p* = 0.003), and CAT (*p* = 0.007) were observed among the experimental groups. When comparing the K3R-treated group to the Ctrl group, morphometric analysis revealed an increase in the diameter of muscle fibers (*p* = 0.011). The levels of IL-6 (*p* = 0.042) and TNF-α (*p* = 0.005) indicated attenuation of the systemic inflammatory response, while reduced S100β levels (*p* = 0.002) were observed in the K3R-treated group, consistent with reduced injury-associated changes. Conclusions: Our findings suggest that K3R may facilitate early functional recovery following sciatic nerve injury, potentially by attenuating oxidative stress and systemic inflammatory responses. However, the precise mechanisms underlying this remain to be elucidated. Future studies should therefore focus on identifying the molecular pathways involved in K3R-mediated recovery, as well as axonal regeneration, Schwann cell activity, and remyelination. Long-term investigations incorporating structural, morphometric, and molecular assessments will be essential to understand its regenerative efficacy and translational potential.

## 1. Introduction

Peripheral nerves play a crucial role in transmitting neural signals between the central nervous system and target organs and tissues. Any interruption in the transmission of messages can lead to temporary or lifelong disability. Certain conditions or disorders, including crush injuries, compression, lacerations, scarring, fractures, iatrogenic traumas, rheumatoid arthritis, diabetes, systemic lupus erythematosus, and Guillain-Barré syndrome, can cause damage to these nerves [1,2]. The reported incidence of traumatic and surgery-related peripheral nerve injuries (PNIs) ranges from 13 to 23 cases per million individuals [3,4]. The most common causes of PNIs in a developing nation like Pakistan include road accidents, stretching or crushing, and penetrating trauma from gunshots and stabbings. These incidents mostly result in direct brain, lower limb, or upper limb injuries such as hand and wrist injuries [5]. A poor prognosis, a long recovery period, and complex repair mechanisms are the hallmarks of PNIs [6,7]. PNI triggers a cascade of pathological processes, including demyelination, oxidative stress, phagocytosis, and inflammation, which disrupt impulse conduction and initiate degenerative changes in the injured nerve. This results in loss of both sensory and motor functions [8], and a persistent state of denervation can lead to muscle atrophy and irreversible disability [9]. Following an injury, the injured nerve undergoes Wallerian degeneration in the distal segment, followed by a regenerative response aimed at restoring axonal continuity and reinnervating target tissues. Regenerating axons extend from the proximal nerve stump toward distal targets within a supportive environment largely orchestrated by Schwann cells. In humans, axonal regeneration is deadly slow, typically proceeding at approximately 1 mm per day, although the rate can vary depending on the type, severity, and location of the injury. Consequently, prolonged regeneration distances may result in delayed or incomplete reinnervation. Functional recovery ultimately depends not only on axonal regrowth but also on the successful reinnervation and restoration of distal target organs, particularly skeletal muscles and sensory structures [10]. Currently, the available intervention system is primarily based on microsurgical suturing and autologous nerve transplantation, supplemented by pharmacological treatments such as B-complex vitamins, mecobalamin, and neurotrophic factors. However, existing therapeutic approaches achieve only anatomical reconstruction and symptomatic support, failing to restore neurological function [11]. In this scenario, there has been increased focus on identifying therapeutically potent, inexpensive, and accessible agents that can restore sensorimotor function following sciatic nerve injury [12]. Natural bioactive compounds appear as a beacon of hope, as they have been serving humans with their positive impact on health ever since. We previously reported that plant extracts exert ameliorating effects on peripheral nerve regeneration in an animal model of sciatic nerve injury [13,14]. Our recent study discloses that Kaempferol-3-O-rutinoside (K3R) can be a potential agent to accelerate the restoration process [15].

K3R, a flavonoid glycoside, is derived from tea, plants, and ancient Chinese medicine. It offers anti-atherosclerotic, anti-diabetic, cerebral, hepatoprotective, anti-cancer, wound-healing, cardioprotective, antiviral, renoprotective, and anti-adipogenic effects [16,17,18]. It diminishes inflammation in macrophages by specifically binding vascular endothelial growth factor C (VEGF-C) [19] and has neuroprotective effects in nervous system disorders [20]. Therefore, the present study aimed to evaluate the potential of K3R to improve sensorimotor functional recovery in mice subjected to sciatic nerve injury.

## 2. Materials and Methods

### 2.1. Compound

The two-dimensional structure of K3R (molecular weight 594.5 g/mol) was generated using ChemDraw 22.0 (Figure 1).

### 2.2. Network Pharmacology

Molecular docking was used to evaluate the interaction strength of K3R with S100β (PDB ID: 3HCM) using the Glide module of Schrödinger Maestro 12.5 (Schrödinger Release 2020-3) [21,22]. The protein was prepared using the Protein Preparation Wizard of Schrödinger, while K3R and the co-crystallized ligand (S45) were prepared using LigPrep in. The receptor grid was generated at the co-crystallized ligand binding site, and docking was performed using the Glide Standard Precision (SP) protocol. GlideScore and protein-ligand interactions were analyzed using Maestro 12.5. The docking protocol was validated by redocking S45, which produced an RMSD of 3 Å compared with the experimental crystallographic confirmation.

### 2.3. Cytotoxicity Assessment (MTT Assay)

K3R cytotoxicity was assessed in HEK-293 cells using the MTT assay as a preliminary in vitro safety evaluation. The main goal of this assay was to validate its safety profile prior to carrying out in vivo experiments. Cells were treated with various concentrations of K3R (25, 50, and 100 µg/mL) to determine whether K3R affects HEK-293 cell survival. A seeding density of 2 × 10^4^ cells/well was maintained for cell growth. The culture plate was placed in a CO_2_ incubator and incubated for 24 h. For treatment, 10 µL of the test sample was mixed with fresh DMEM (Dulbecco’s Modified Eagle Medium). After adding the medium, cells were incubated for an additional 48 h. Following incubation, 5 mg/mL MTT solution was added to each well, where living cells reduced it to insoluble purple formazan crystals. A 150 µL volume of DMSO (Dimethyl Sulfoxide) was added to the wells. The optical density (OD) was measured at 560 nm [23].

### 2.4. Animals

Healthy 8–10-week-old albino mice (BALB/C) weighing 25–30 g were obtained from the Animal Research Station, Department of Physiology, Government College University, Faisalabad. After 5 days of acclimatization, the animals were randomly divided into three groups (*n* = 8/group). Sham (Group 1), Control (Group 2), and K3R (Group 3; administered K3R 2.5 mg/kg via intraperitoneal injection). They were housed in appropriate cages. The instructions given by the International Association for the Study of Pain (IASP) were followed for the use of experimental animals in pain research. Animals had free access to food and water and were housed in separate cages at 23 ± 2 °C under a 12-h light/dark cycle. After the trial, blood was obtained for biochemical and molecular analyses.

### 2.5. Sciatic Nerve Injury Induction

Following acclimatization, sciatic nerve injury was induced in the right hind limb as previously described [24]. Briefly, mice were anesthetized by intraperitoneal injection of 70 mg/kg ketamine and 5 mg/kg xylazine. The injury site was shaved. The sciatic nerve was exposed. The left hind limb (Contralateral) of the mice remained unoperated, and the sciatic nerve in the right hind limb (Ipsilateral) was compressed with a pair of forceps for 15 s. A transparent ring appeared at the injury site, which indicated nerve damage. The wound was closed with 2–4 sutures, and povidone–iodine was applied to prevent infection. Mice were kept on the hot pad after surgery. Mice in the sham group were subjected to the same surgical procedure as other injury groups, that include anesthesia, incision of the skin, and exposure of the sciatic nerve; however, the sciatic nerve remained intact without induction of crush injury. The surgical wound was closed with sutures. The Ctrl group underwent sciatic nerve injury but did not receive any treatment.

### 2.6. Drug Administration

Twenty-four mice were randomly divided into three groups (*n* = 8 per group). This sample size was selected in accordance with a previous study using similar sciatic nerve crush models. The groups were as follows: (1) SNI + Ctrl (sciatic nerve injury + 5% DMSO): animals subjected to sciatic nerve injury and receive no treatment; (2) Sham (5% DMSO): animals in sham group were not subjected to injury and do not receive a treatment, (3) SNI  +  K3R (sciatic nerve injury  +  K3R): animals subjected to sciatic nerve injury received K3R (2.5 mg/kg) via intraperitoneal injection (IP) once daily for 12 consecutive days. Treatment administration was not performed under blinded conditions.

### 2.7. Physical Parameters

#### Body Mass and Diet Intake

In this experiment, each mouse’s body mass was measured daily to evaluate the effects of the treatment. Daily diet intake was recorded at a predetermined time during the light/dark cycle to monitor changes associated with injury and treatment [25].

### 2.8. Behavioral Analyses

Sensory and motor function tests were performed before and after the injury. Behavioral analyses were carried out in a blinded manner.

#### 2.8.1. Hotplate Test

For the assessment of sensory function, a hotplate test was used. The hotplate’s temperature (SCILOGEX Ms7-H550-S LED digital 7 × 7 Hot plate, SCILOGEX, Rocky Hill, CT, USA) was set at 56 ± 2 °C, and the mouse was placed on the heated surface. The mouse abruptly withdrew its ipsilateral hind paw, licked it, or jumped, which defined the response time known as hot plate latency (HPL). A stopwatch was used to measure the latency period, called the withdrawal reflex latency (WRL). A cutoff time of 30 s was applied if the response latency in mice exceeded this duration. A total of three measurements were taken, and the mean latency was used for statistical analysis [26].

#### 2.8.2. Pinprick Test

The pinprick test was used to assess sensory functional recovery following sciatic nerve injury. Briefly, the lateral plantar surface of the mouse’s hind paw was divided into five regions, and it was placed in a wire-grid cage. An Austerlitz insect pin of size “000” was gently used to prick each area, and a positive response was recorded after a swift paw withdrawal. The experiment started at the heel ‘A’ and ended at the outermost toe ‘E’ of the affected paw. No response was scored as “0,” and “1” indicated a positive response [27]. Therefore, a score of 5 reflects complete sensory recovery, and a score of “0” signifies total functional loss, while a score below five suggests incomplete recovery.

#### 2.8.3. Sciatic Functional Index (SFI)

After nerve injury, the SFI test was used for the evaluation of motor function recovery. The hind paws of mice were dipped in ink, and they were allowed to walk on a wooden walkway (50 cm long); its floor was covered with white chart paper to obtain footprints. The following formula was used for the calculation of SFI:

$$SFI=\left(-38.3\times -\frac{EPL-NPL}{NPL}\right)+\left(109.5\times \frac{ETS-NTS}{NTS}\right)+\left(13.3\times \frac{EIT-NIT}{NIT}\right)-8.8$$

The print length (PL: Distance between the tip of the third toe and the heel), intermediate toe spread (IT: Distance between the second and fourth toe), and toe spread (TS: Distance between the first and fifth toe) were measured from footprints. For the calculation of SFI, the measurements from the experimental paw (EPL, ETS, EIT) were compared with those of the normal paw (NPL, NTS, NIT) [28].

#### 2.8.4. Grip Strength Test

Grip strength was assessed as a measure of motor functional recovery using a grip strength apparatus (Bioseb, Chaville, France). For this, each mouse was allowed to grasp the grid bar of the strength meter and was gently pulled backward until it released the grid. The gripping force of the paws was measured in Newtons (N). Three readings were obtained from both contralateral and ipsilateral hind limbs, and the mean of these readings was used for statistical analysis [29].

### 2.9. Measurement of Blood Glucose Levels

Blood glucose levels were measured to monitor systemic metabolic changes following sciatic nerve damage. Blood was collected from the tail vein. At different time points during the experiment, blood glucose levels in mice were assessed using a glucometer (Accu-Chek, Corydon, IN, USA) according to the manufacturer’s instructions [30].

### 2.10. Evaluation of Biochemical Markers

At the end of the experiment, blood samples were collected and centrifuged to obtain serum for biochemical analyses. These assays were conducted without blinding.

### 2.11. Assessment of Oxidative Stress

#### 2.11.1. Measurement of Total Antioxidant Capacity (TAC)

The biological system has antioxidant capacity that neutralizes free radicals and the associated physiological damage to the body. Serum TAC was measured to evaluate the overall antioxidant capacity of the body. In this process, the ABTS solution turned green as a result of a reaction in which hydrogen peroxide oxidized ABTS (2,2′-azino-bis (3-ethylbenzothiazoline-6-sulfonic acid)) under an acidic environment. When the serum sample was added to this solution, the color intensity of the solution decreased. The presence and absence of antioxidants directly influence this process. A chemistry analyzer (BIOLAB310, URIT, Guilin, China) was used to measure the absorbance of the solution at 650 nm. Vitamin C was used as a standard for this analysis. The TAC was expressed in mmol/L of Vitamin C [31].

#### 2.11.2. Measurement of Total Oxidant Status (TOS)

The body’s overall oxidative state is known as total oxidant stress. It may harm body systems due to an imbalance between antioxidant and oxidant defenses [32]. After nerve damage, the total oxidant status (TOS) of each group was calculated from serum to evaluate oxidative stress. To determine serum oxidant level, a spectrophotometer (BIOLAB 310) was used. The substrate used in the assay was a combination of dianisidine–ferrous ion. A colorful complex was formed after blending ferric ions and xylenol orange in an acidic environment. In this mixture, the color intensity was directly proportional to the concentration of the oxidants. An automated analyzer (BIOLAB 310) was used to measure the absorbance of the mixture at 560 nm. Hydrogen peroxide was used to calibrate this analysis. H_2_O_2_ was used as the standard for calibration, and the results were reported as µM H_2_O_2_ Eq./L [33].

### 2.12. Assessment of Malondialdehyde (MDA) Activity

This assay uses the Thiobarbituric method to measure the quantity of MDA. The serum sample (100 μL) was diluted with distilled water (900 μL), then mixed with the TBA reagent (500 μL). The mixture was then allowed to cool to 100 °C for 1 h and centrifuged for 10 min at 4000 rpm. Using a spectrophotometer (UH5300; HITACHI, Tokyo, Japan), the supernatant’s absorbance was measured at 534 nm. The MDA concentration was calculated using the silence factor (105 × 1.56) and expressed as mmol/L [34].

### 2.13. Measurement of Catalase Activity

The body’s ability to neutralize oxidative impairment was evaluated by measuring the level of catalase. The catalase activity was measured according to the previously reported protocol. Briefly, 50 μL of the sample was added to distilled water and allowed to incubate for three minutes. After this, Hydrogen peroxide (H_2_O_2_) was added to the mixture. 500 μL of dichromate was added after this step. The mixture was heated in a water bath and then cooled at ambient temperature, and centrifugation was performed at 25,000 rpm on the samples. A chemical analyzer (Biolab 310: URIT, Guilin, China) was used to take measurements at a wavelength of 545 nm [35].

### 2.14. Assessment of S100β Protein Level

The level of S100β protein was measured using an ELISA kit (LOT: 202505; Glory Science, Shanghai, China). After adding 10 µL of the samples, the standard was added to the wells. Then, each well received HRP-conjugate and 40 µL of the sample diluent. After using an adhesive strip to seal the plate, it was incubated for 60 min at 37 °C. The wells underwent five successive washes after incubation. Chromogen solutions A and B were dispensed into the wells after aspirating the previous solution. After this step, the plate was incubated. After adding the stop solution, the color changed from blue to yellow. After 15 min, the optical density (O.D.) at 450 nm was measured using a microtiter plate reader (URIT-660, Guilin, China) [36].

### 2.15. Estimation of Inflammatory Biomarkers

In the serum of mice, the level of IL-6 (interleukin-6) and TNFα (Tumor necrosis factor-α) (proinflammatory cytokines) was measured using ELISA kits (BT LAB, Shanghai, China). TNFα (Cat. No. E0117Mo) and IL-6 (Cat. No. E0049Mo) were measured using an ELISA according to the manufacturer’s instructions. In each well, 40 µL of serum sample and 10 µL of anti-IL-6 antibody were added; after that, 50 µL of streptavidin-HRP was added. A 60 min incubation at 37 °C was maintained for the well plate. 300 µL of wash buffer was used for five consecutive washes; each rinse lasted between 30 s and 1 min. Substrate solutions A and B were added to the wells, and then, under a controlled environment, the plate was incubated for the development of color. In each well, stop solution was added at a volume of 50 µL to end the enzymatic reaction. The color instantly changed from blue to yellow. At 450 nm, the O.D. of the samples was recorded using an ELISA plate reader. The O.D. was measured within 10 min after adding the stop solution. Using an ELISA kit, the same procedure was performed to quantify TNF-α. Each sample’s protein concentration is reported in pg/mg [37].

### 2.16. Analysis of Blood Cell Profile

The blood was collected from the mice in EDTA-anticoagulated vials. The concentration of hemoglobin and the number of other blood cells (WBCs, monocytes, neutrophils, lymphocytes, eosinophils, and platelet count) were determined by using a hematology analyzer (Swelab AlfaLyse, Stockholm, Sweden) (LOT: 2407-218). These parameters were measured to assess immune and systemic inflammatory responses [38].

### 2.17. Effect of K3R on Muscle Fiber Morphology

Tibialis anterior muscles were harvested from both hind limbs at the end of the trial. 10% neutral-buffered formalin was used for fixation of tissues for 24–48 h. For the removal of fixative, tissues were washed in PBS overnight. Tissues were dehydrated in a series of alcohols. In the next step, blocks were made by embedding tissues in paraffin. These blocks were then cut using a microtome (Leica RM2125: LEICA, Deer Park, IL, USA). Sections were placed on slides, covered with a coverslip, and stained with the H&E (Hematoxylin and Eosin) method. A microscope (XSZ 107BN, Hefei, China) was used to observe morphometric changes in the ipsilateral and contralateral muscles at 40× magnification. An Optika B1 camera (OPTIKA, Ponteranica, Italy) was used to capture pictures. For each mouse, 5 images were analyzed. Lastly, a specific region of the image was selected to measure each cross-sectional area of the muscle fiber using ImageJ (version 1.52). The mean value for each fiber was calculated from all images and then compared across the groups [39].

### 2.18. Statistical Analyses

Results were analyzed using GraphPad Prism 8.4.2 and were presented as mean ± SEM. Group means at different time points were compared using Tukey’s and Dunnett’s multiple comparisons tests and analysis of variance (ANOVA). For every observation, a statistically significant value was defined as *p* < 0.05.

## 3. Results

### 3.1. Docking Score and Validation of the Docking Protocol

K3R was docked into the human S100β binding site (PDB ID: 3HCM), yielding a favorable GlideScore of −5.672 kcal/mol. The score provides a relative measure of predicted binding when compared with other compounds using the same docking protocol. The reference ligand S45 showed hydrophobic interactions with Ala83, Leu60, and Met79 (Figure 2a,b). Redocking of S45 yielded an RMSD of 3.3 Å relative to the crystallographic pose, indicating a moderate deviation and partial preservation of the native orientation (Figure 2c).

K3R occupied the S100β binding pocket through hydrophobic and polar contacts, including π-alkyl and alkyl interactions with Leu44 and Val52, hydrogen bonds with Glu45 and Thr59, and a carbon-hydrogen bond with Val52. Met79 contributed a π-sulfur interaction, while the 3D pose confirmed these residues as the main interacting residues (Figure 2d).

### 3.2. Impact of K3R on the Viability of HEK-293 Cells

The MTT assay was used to assess K3R’s cytotoxicity. High viability was observed in cells treated with K3R at 25, 50, and 100 μg/mL, suggesting negligible cytotoxicity. Cell survival was maintained above 95% in all treatment groups, despite a slight concentration-dependent decline in cell viability with increasing K3R concentration. The assay’s cytotoxic sensitivity was confirmed by the positive control, which consisted of cells treated with doxorubicin and showed a significant decrease in cell viability. K3R treatment significantly reduced cell death and preserved cellular viability, which was much higher than in the doxorubicin-treated group. These results indicate that K3R exhibited low cytotoxicity within the tested concentration range (Figure 3).

### 3.3. Impact of K3R on Body Mass and Diet Intake

Throughout the experimental period, body mass and diet intake were monitored in each group. Two-way ANOVA showed a significant difference in body mass (* *p* = 0.035) and no significant difference in diet intake (*p* = 0.965) between the K3R-treated and Ctrl groups following the induction of nerve damage and treatment (Figure 4a,b).

### 3.4. Effect of K3R on Recovery of Sensory Function

Following sciatic nerve damage, the sensory functional recovery was evaluated using the pinprick and hotplate tests (Figure 5a,b). Two-way ANOVA showed that the pinprick score in the K3R-treated group was significantly reduced (*** *p* < 0.001) post-injury relative to the Ctrl and sham groups. According to two-way ANOVA the paw withdrawal latency from the hot plate surface showed a statistically significant reduction (*** *p* < 0.001) in the K3R-treated group. These results confirm that K3R accelerates the recovery of sensory threshold after sciatic nerve injury.

### 3.5. Effect of K3R on Motor Function Recovery

Two-way ANOVA showed significant differences in motor function recovery. In the current scenario, mice in the K3R-treated group showed a noticeable improvement in their movement patterns, as indicated by SFI data (*** *p* < 0.001) after injury. Similarly, the K3R-treated group exhibited a significant recovery (*** *p* < 0.001) in paw’s gripping power in comparison to the Ctrl and sham groups (Figure 6a,b).

### 3.6. Effect of K3R on Glycaemic Level

Two-way ANOVA showed no significant difference in glucose levels between the K3R-treated, Ctrl, and sham groups (*p* = 0.364), showing that K3R treatment had no significant effect on blood glucose levels (Figure 7).

### 3.7. Effect of K3R on Oxidative Stress Markers

One-way ANOVA showed significant differences in oxidative status among the groups. K3R treatment significantly altered several markers of oxidative status. A significant reduction was observed in the Ctrl group in TAC as compared with the sham group, whereas there was a significant increase in the TAC value of the K3R-treated group compared to the Ctrl group (*** *p* < 0.001) (Figure 8a). TOS was significantly higher in the Ctrl group than in the sham group. In contrast, the K3R-treated group had a significantly lower TOS than the Ctrl group (*** *p* < 0.001), indicating attenuation of oxidative stress (Figure 8b). Likewise, a significant increase in MDA levels was observed in the Ctrl group as compared with the sham group. The K3R-administered group exhibited a lower MDA level than the Ctrl group (** *p* = 0.003), suggesting reduced lipid peroxidation (Figure 8c). Catalase activity was decreased significantly in the Ctrl group as compared to the sham group. Whereas a significant increase in catalase activity was observed in the K3R-treated group relative to the Ctrl group (** *p* = 0.007), indicating increased catalase activity (Figure 8d).

### 3.8. Impact of K3R on S100β Protein Level

As a biomarker associated with glial responses and neural injury, S100β protein levels were measured using ELISA in mouse serum samples. After injury, serum S100β concentration was significantly higher in the Ctrl group in comparison to the sham group. According to one-way ANOVA, the K3R-treated group’s serum S100β levels significantly decreased (** *p* = 0.002) in contrast to the Ctrl group, consistent with reduced neural injury and altered glial responses (Figure 9).

### 3.9. Influence of K3R on Inflammatory Marker Profiles

Following injury, TNF-α and IL-6 (proinflammatory cytokines) were measured to determine the inflammatory response. The Ctrl group showed higher serum concentrations of both TNF-α and IL-6 than the sham group, indicating a sustained inflammatory response. In contrast, the K3R-treated group showed significantly reduced TNF-α (** *p* = 0.005) and IL-6 (* *p* = 0.042) levels in serum relative to the Ctrl group, as shown by one-way ANOVA indicating attenuation of systemic inflammation following injury (Figure 10a,b).

### 3.10. Effect of K3R on Hematological Indices

One-way ANOVA showed that there was a significant increase in Hb level in the K3R-treated group (** *p* = 0.010) as compared to the Ctrl group. A non-significant difference (*p* = 0.237) was observed in platelet count following K3R treatment, and a significant change in WBC count was observed (* *p* = 0.014) (Figure 11a–c).

### 3.11. Impact of K3R on Differential Blood Count

According to one-way ANOVA, the neutrophil (*p* = 0.704), lymphocyte (*p* = 0.813), monocyte (*p* = 0.630), and eosinophil (*p* = 0.296) counts exhibited no significant change between the K3R-treated and the Ctrl groups (Figure 12a–d).

### 3.12. Histopathology

One-way ANOVA showed significant differences in tibialis muscle fiber measurements. A significant increase in the value of tibialis fiber count was recorded in the K3R-treated group (* *p* = 0.011) relative to the Ctrl group after the comparison of contralateral and ipsilateral muscle fibers. These findings show that the K3R-treated group helps to maintain normal muscle structure and reduce muscle degradation (Figure 12a). Likewise, a statistically significant difference was observed between the contralateral and ipsilateral muscle cross-sectional areas in the K3R-treated group (** *p* = 0.004), indicating improvement in muscle cross-sectional area following treatment (Figure 13a–c).

## 4. Discussion

Peripheral nerves are vulnerable to a variety of traumas, ranging from minor compression and stretching injuries to severe cuts that result in major nerve abnormalities. Due to decreased dexterity, susceptibility to cold, poor sensory and motor function, and potential pain issues, a nerve injury significantly affects the patient’s day-to-day functioning [40]. Although various therapeutic approaches, including tissue engineering, autologous or allograft nerve transplantation, and surgical repair, are available, complete functional recovery following peripheral nerve injury remains challenging. The functional recovery of peripheral nerves depends on several factors, including the injured nerve’s cross-sectional area, the length of the nerve gap, and cellular and molecular components such as Schwann cells, kinases, growth factors, and inflammatory factors [41].

Vitamin B12 is commonly used to treat peripheral neuropathy since it is known to be essential for neuroprotection [42]. However, the use of vitamin B12 for treating nerve injury is limited due to its adverse effects, including diarrhea, skin irritation, rash, allergic reactions, and even anaphylactic shock [43]. Interestingly, humans and mice showed a key healing time of around 35 days following mild nerve damage [44]. In severe injuries, the time required for nerve repair in humans ranged from months to years, depending on the severity and distance of the injuries. This implies that the inability to repair neuromuscular junctions may be due to the delayed peripheral nerve recovery following an injury, and that there exists a narrow time period during which reinnervation might restore connection with denervated end plates [45]. Therefore, it indicates the need for a therapeutic strategy that can accelerate peripheral nerve recovery and restore sensorimotor function following peripheral nerve injury. Research studies on flavonoids indicate that this chemical family has a significant neuroprotective effect [46,47]. Flavonoids have been shown to enhance neurological recovery in animal models of sciatic nerve injury [48], though limited knowledge exists regarding the pharmacological mechanisms underlying these effects. In this context, we aimed to explore the neuroprotective potential of the flavonoid K3R against our well-established sciatic nerve injury mouse model.

K3R is a flavonoid glycoside found in several plant species and has been reported to exhibit antioxidant and anti-inflammatory properties. Numerous biological functions of K3R have been documented, including antioxidant activity, liver and gallbladder protection, antibacterial, anti-inflammatory, uric acid inhibition, anti-glycation, kidney stone prevention, and wound healing [18,20,49,50,51,52]. K3R and its derivatives have shown neuroprotective effects across a range of neurodegenerative disorders. Its well-known anti-inflammatory and antioxidant properties enhance its neuroprotective effects. It reduces MDA levels, enhances glutathione (GSH) and superoxide dismutase (SOD) activity, and prevents excessive reactive oxygen species (ROS) generation [53].

K3R treatment was associated with a significant change in body mass, whereas food consumption remained unchanged (*p* = 0.965). After sciatic nerve injury, the SFI is a useful parameter for evaluating motor functional recovery. This is achieved by analyzing mouse footprints while they walk [13]. Functional recovery improved naturally over many weeks, leading to enhanced motor performance in animals [54]. In the present study, K3R treatment was associated with progressive improvement in locomotor function following sciatic nerve injury. A significant recovery in walking pattern was observed on day 6 after injury, with Ctrl vs. K3R (*** *p* < 0.001), and on day 11, with Ctrl and K3R (* *p* = 0.032) following nerve crush. The gripping power of mice’s paws was noticeable (*** *p* < 0.001) in Ctrl (Ipsi) vs. K3R (Ipsi) on day 4 post-injury. There was a significant (* *p* = 0.012) difference in grip strength force of mice on day 11 after injury in Ctrl (Ipsi) vs. K3R (Ipsi).

Following sciatic nerve crush damage, the K3R-treated mice exhibited both initial and complete sensory responses more quickly than the sham and Ctrl groups. There was a significant difference seen in pinprick score between Ctrl and K3R on day 3 after the injury (* *p* = 0.019). A significant difference was observed between Ctrl and K3R on day 5 (** *p* = 0.009). On day 9, recovery became more pronounced and was significantly greater in Ctrl than in K3R (*** *p* < 0.001). On day 12, recovery was significantly greater in Ctrl than in K3R (* *p* = 0.045). The paw withdrawal latency indicated a significant difference between Ctrl and K3R on days 5, 9, and 12 after the injury (*** *p* < 0.001). These sensorimotor assessments showed that the K3R-treated group showed faster functional recovery than the Ctrl and sham groups following nerve damage.

K3R treatment showed no significant effect on blood glucose levels (*p* = 0.734) compared with the Ctrl and sham groups, indicating that changes in systemic glucose homeostasis are unlikely to be the mechanism underlying its neuroprotective benefits in sciatic nerve injury. Furthermore, the compound did not negatively impact metabolic function throughout the treatment period, as seen by the maintenance of normal glucose levels. Furthermore, this study demonstrates that the TAC value (*** *p* < 0.001) improved and the TOS level (*** *p* < 0.001) decreased in the K3R group compared to the Ctrl and sham groups. The significant reduction in MDA and increase in catalase activity further support the antioxidant activity of K3R following sciatic nerve injury. Hence, K3R acts as an antioxidant by directly scavenging free radicals.

In studies of sciatic nerve injury, S100β, a well-known marker of Schwann cells, is frequently used to evaluate glial responses and neural damage [55]. In this study, the level of S100β significantly decreases in Ctrl vs. K3R (** *p* = 0.002). The reduction in S100β levels may indicate decreased glial activation and neural injury, suggesting that K3R supports a favorable post-injury environment. Thus, K3R has a regulatory effect. At the site of injury as well as in the compartments of the nervous system, inflammatory elements are distributed as a result of sciatic nerve injury [56]. This study demonstrates that TNF-α levels decrease significantly in Ctrl vs. K3R (** *p* = 0.005). The level of IL-6 also decreases in Ctrl vs. K3R (* *p* = 0.042). The decrease in these cytokine levels indicates attenuation in injury-related inflammation, which may be favorable for nerve recovery, as uncontrolled or prolonged inflammation can impair functional recovery and axonal regrowth.

Comparison of contralateral and ipsilateral tibialis anterior slides showed that the behavioral findings were supported by histopathological analysis. Following the measurement of the cross-sectional area using ImageJ, each muscle fiber was assessed. As compared to the Ctrl group, which had substantial alterations in the cross-section of contralateral and ipsilateral muscle fibers, the evaluation of the cross-sectional area of muscle fibers between the treated group’s ipsilateral and contralateral muscles was determined to be non-significant. This shows that the nerve may begin to transmit impulses to the afflicted muscle nearly normally. Our findings showed that K3R protects muscle fibers from cellular death and improves functional behavioral recovery [57,58].

Although the present findings provide important insights into the potential role of K3R in accelerating functional recovery following sciatic nerve injury, it has several limitations that should be noted. Since K3R was administered systemically in the current study, future research should assess the effectiveness of local delivery to ascertain its direct effects at the site of injury. Furthermore, the 12-day observation period was intended to record the early phase of post-injury recovery. Extended follow-up is warranted to assess the sustainability of the observed therapeutic effects and contribution to long-term functional restoration. While the present work demonstrated significant improvements in sensorimotor performance and muscle function, direct evaluation of nerve regeneration was not performed in this study. Future investigation should therefore incorporate comprehensive histological, morphometric, and molecular analyses of the injured sciatic nerve, including assessment of axonal density, fiber diameter, myelin thickness, g-ratio, Schwann cell responses, and local inflammatory and oxidative stress status. As a result, the accelerated functional recovery observed in this study should be considered indirect evidence supporting enhanced regenerative processes. The beneficial effects of K3R may be mediated, at least in part, through modulation of oxidative stress and inflammatory pathways; however, the precise molecular mechanisms remain to be elucidated. Future investigations should also examine dose–response, the underlying signaling pathways involved in neural repair, and validate regenerative outcomes using molecular markers of peripheral nerve regeneration.

## 5. Conclusions

Our findings suggest that K3R may enable early functional recovery following sciatic nerve injury in mice, potentially through the modulation of oxidative stress and inflammatory responses. These observations indicate the therapeutic potential of K3R as a promising candidate for improving functional outcomes during the early stages of peripheral nerve repair. However, the precise mechanisms underlying this effect remain to be elucidated. Future studies should therefore focus on identifying the molecular pathways involved in K3R-mediated recovery, as well as axonal regeneration, Schwann cell activity, and remyelination. Long-term investigations incorporating structural, morphometric, and molecular assessments will be essential to understand its regenerative efficacy and translational potential.

## Figures and Tables

**Figure 1:**
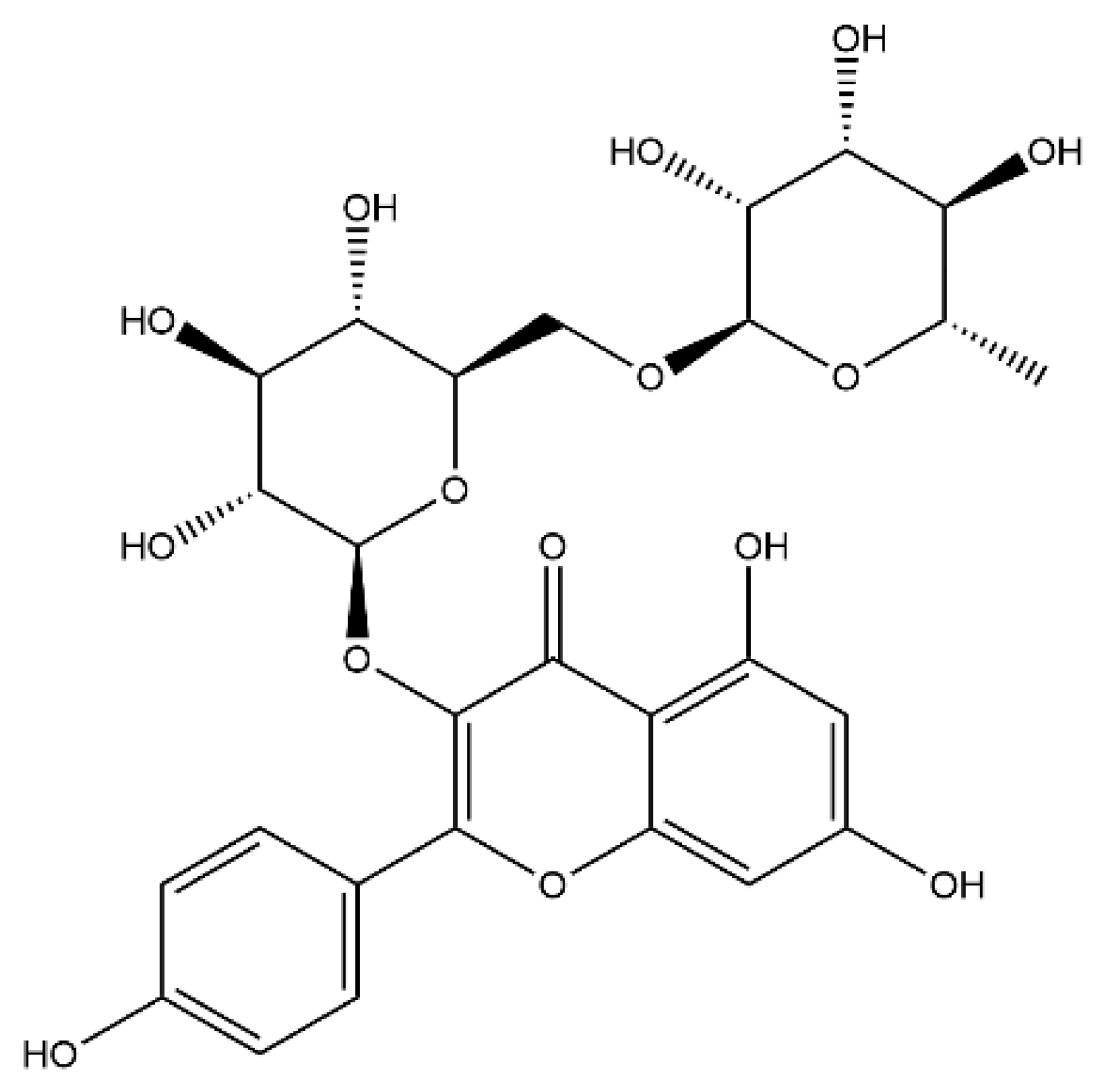
Chemical Structure of K3R.

**Figure 2:**
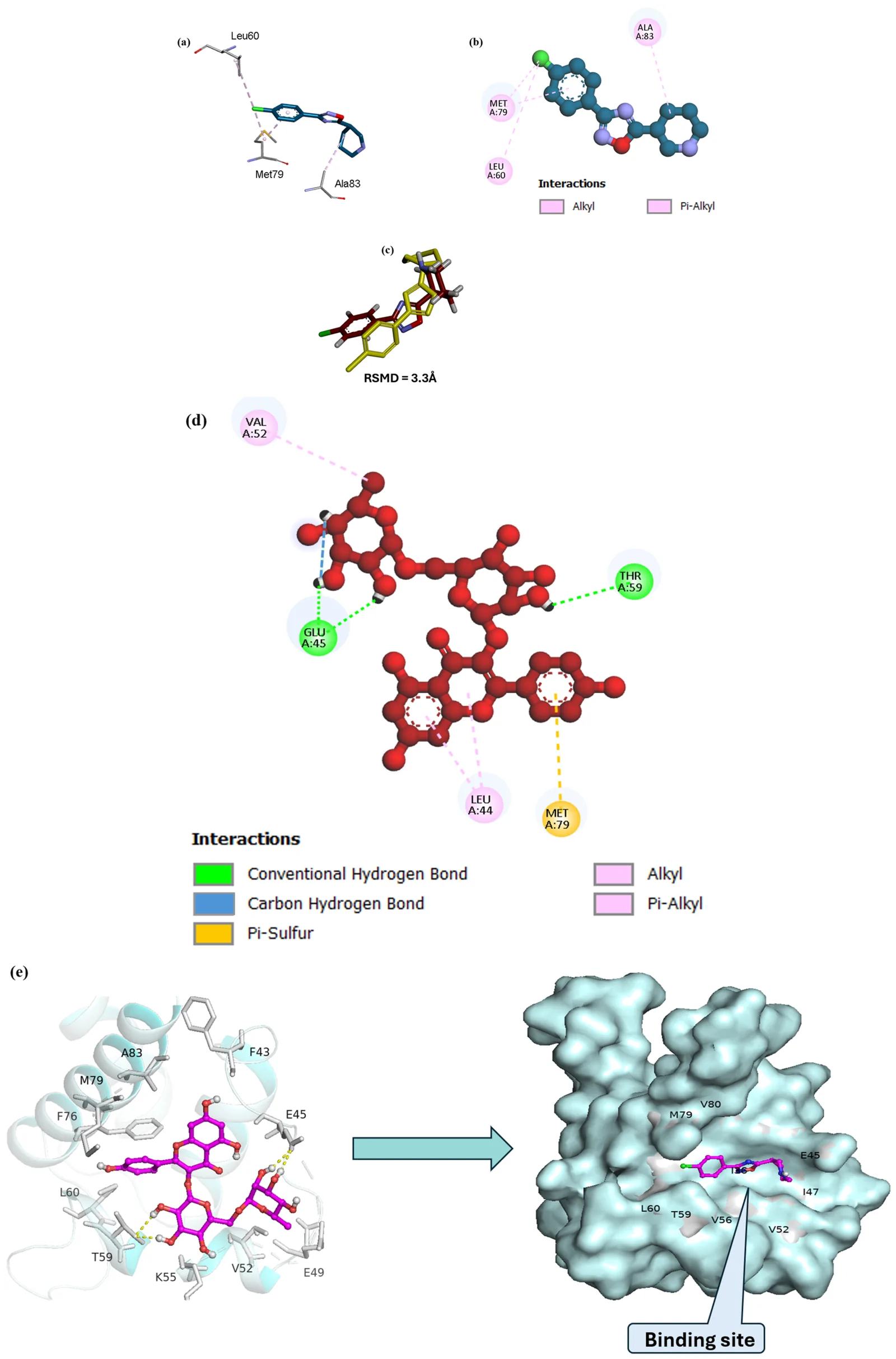
(**a**–**c**): The co-crystallized ligand S45 (CCL) docking and validation studies in the binding site of S100β. (**a**) CCL interactions with Leu60, Met79, and Ala83 are represented in three dimensions; (**b**) alkyl and π-alkyl contacts are illustrated in a two-dimensional interaction map; (**c**) the docked and crystallographic ligand conformations are superimposed (RMSD = 3.3 Å). (**d**) Molecular Interactions of K3R with S100β: K3R’s two-dimensional interaction profile in the binding site of S100β, exhibiting standard hydrogen bonds with Glu45 and Thr59, a carbon-hydrogen bond with Val52, alkyl and π-alkyl interactions with Val52 and Leu44, as well as a π-sulfur contact with Met79. (**e**) K3R’s three-dimensional location inside the binding pocket of S100β. The ligand is depicted on the molecular surface (**right**) and inside the protein cavity (**left**). The binding area is surrounded by the residues Phe43, Glu45, Ile47, Glu49, Val52, Lys55, Val56, Thr59, Leu60, Phe76, Met79, Val80 and Ala83.

**Figure 3:**
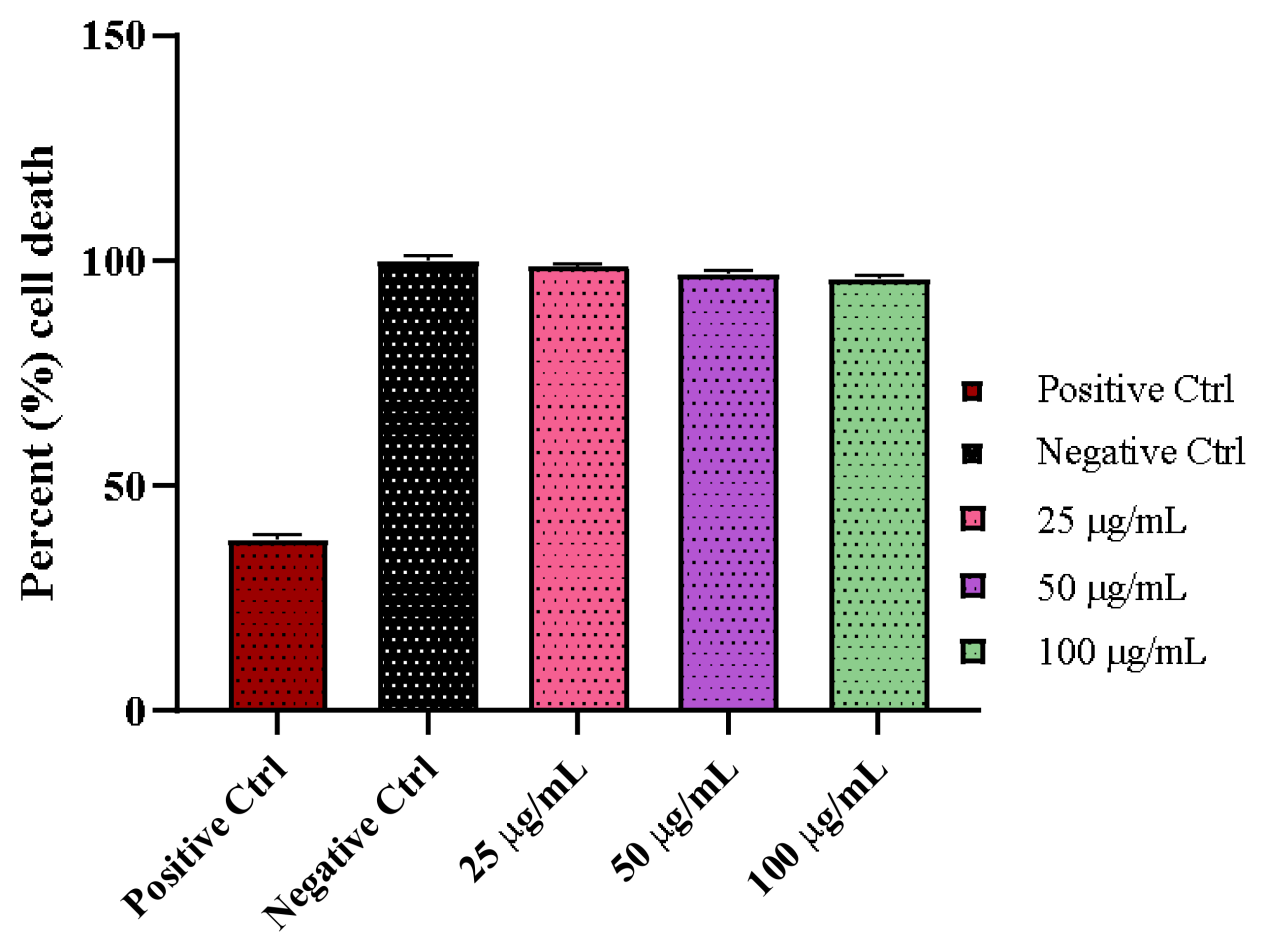
The cytotoxic effect of K3R on HEK-293 cells: Cytotoxic effect of K3R assessed by MTT assay. Cells were treated with K3R at different concentrations (25, 50, and 100 μg/mL), while doxorubicin and DMSO served as positive and negative controls, respectively. K3R showed considerably less cytotoxicity than the doxorubicin-treated group and maintained cell viability over 95% at all tested concentrations. One-way ANOVA showed a significant difference (*p* < 0.001).

**Figure 4:**
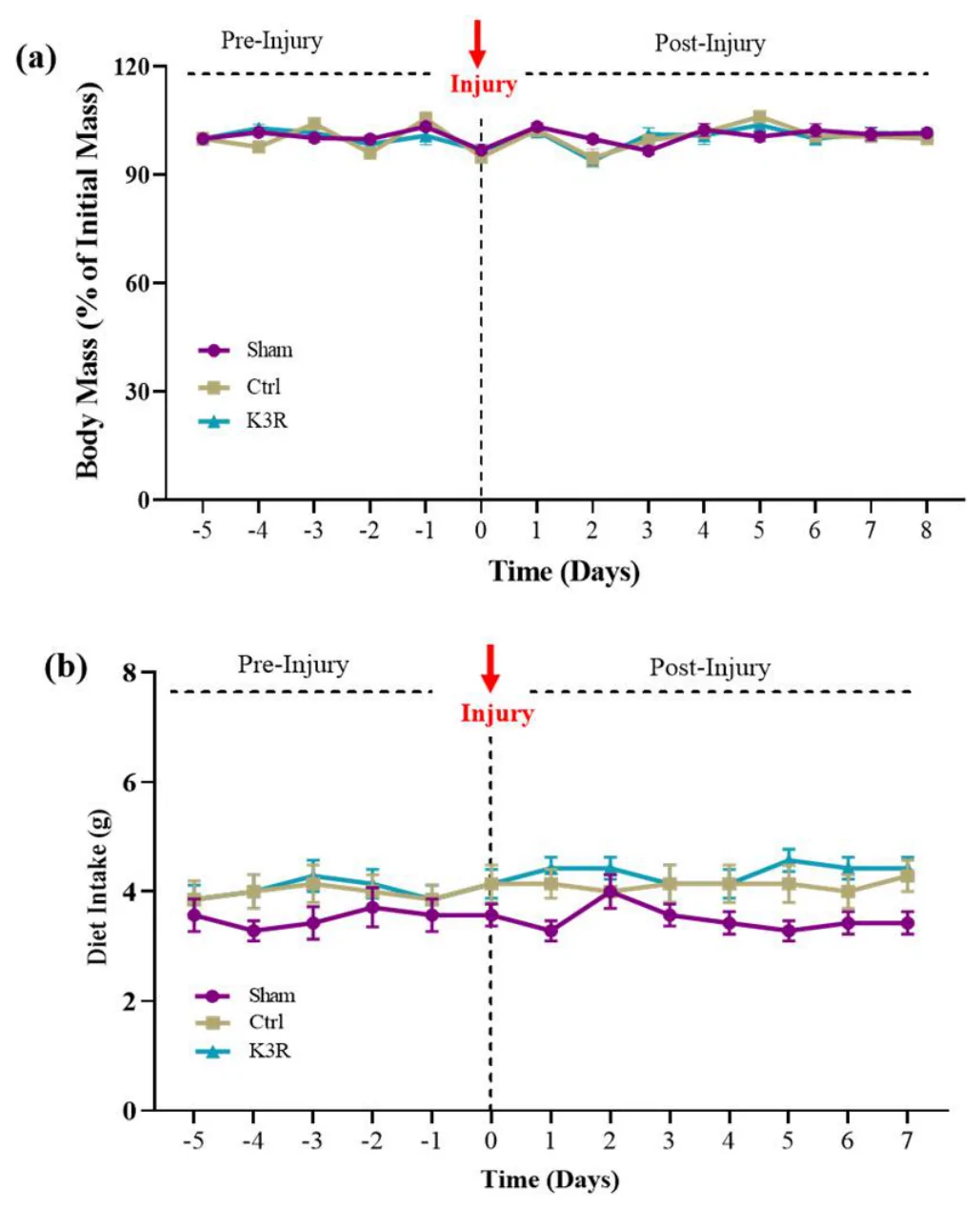
Impact of K3R on body mass and diet intake: Data were analyzed using two-way ANOVA followed by Tukey’s multiple comparisons test. No significant difference was observed in (**a**) body mass or (**b**) diet intake between the K3R-treated and the Ctrl groups at any time point before and after sciatic nerve injury. Data are represented as mean ± SEM (*n* = 8 per group).

**Figure 5:**
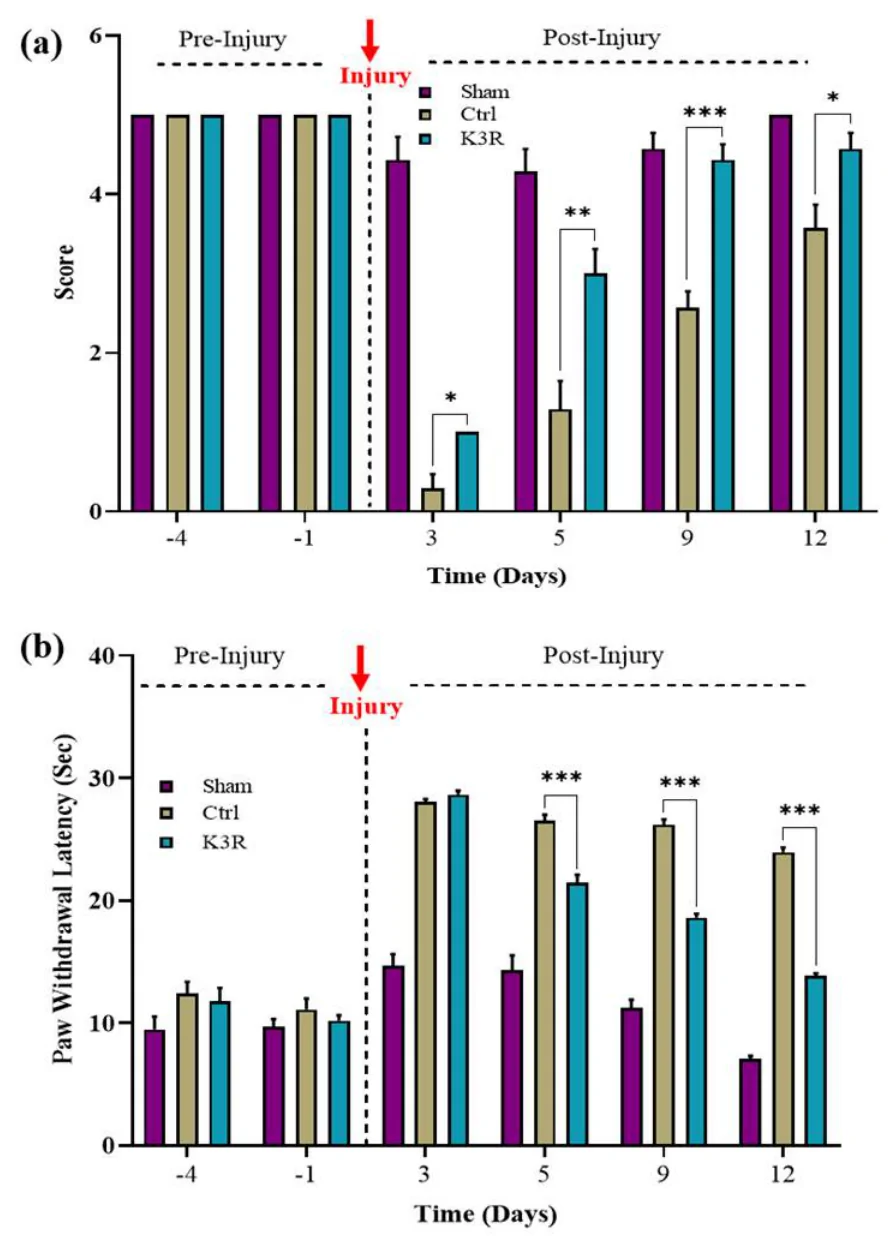
Effect of K3R on sensory function recovery: Statistical significance among the experimental groups was assessed using two-way ANOVA followed by Tukey’s multiple comparisons test. (**a**) The K3R-treated group showed significantly higher pinprick response scores on days 3 (* *p* = 0.019), 5 (** *p* = 0.009), 9 (*** *p* < 0.001), and 12 (* *p* = 0.045) after injury relative to the Ctrl group. (**b**) Paw-withdrawal latency in response to thermal stimulation was significantly reduced in the K3R-treated group on days 5 (*** *p* < 0.001), 9 (*** *p* < 0.001), and 12 (*** *p* < 0.001) compared with the Ctrl group. Values are expressed as mean ± SEM (*n* = 8 per group).

**Figure 6:**
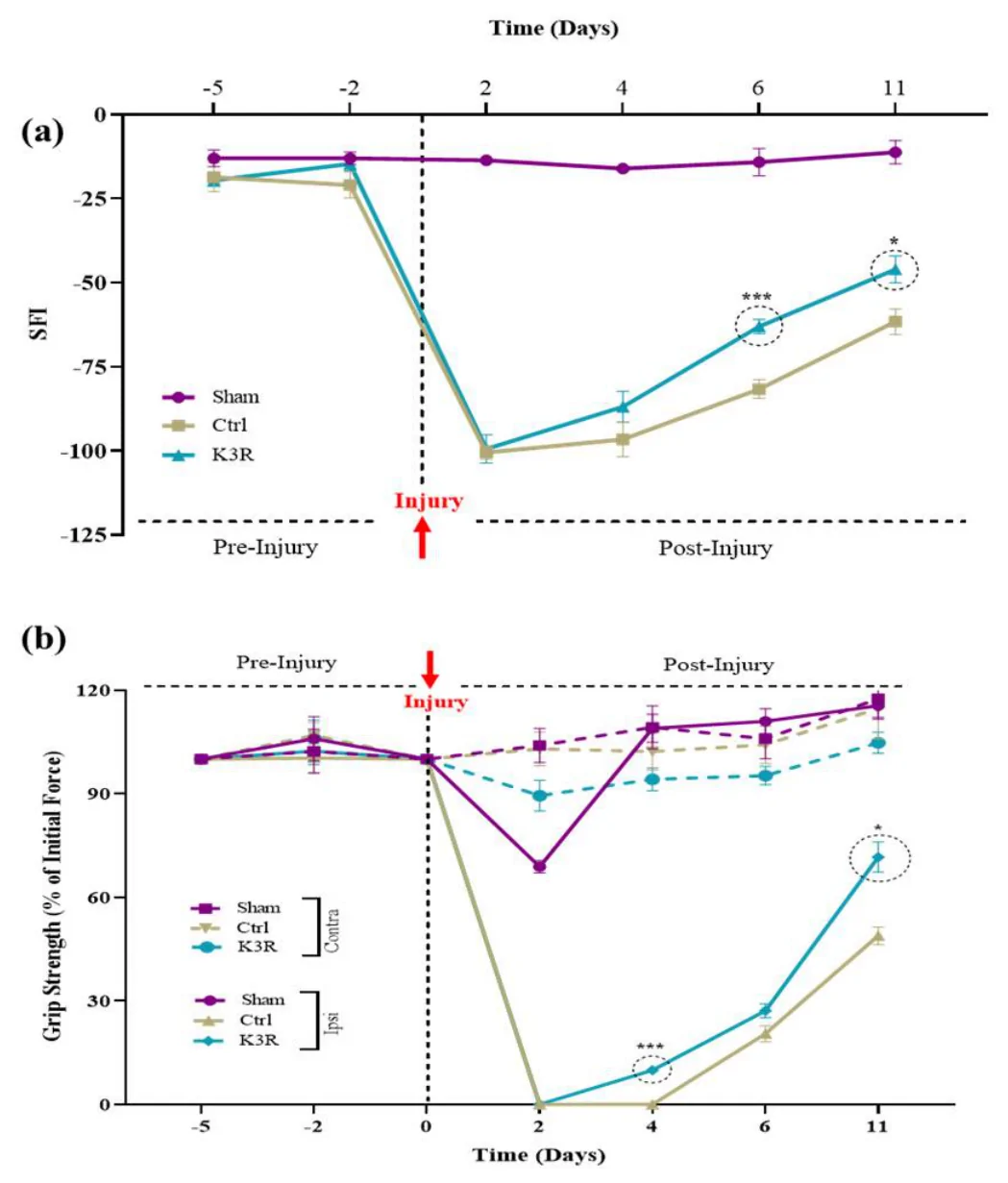
Effect of K3R on motor function recovery: Differences between groups were evaluated using two-way ANOVA followed by Tukey’s multiple comparisons test. (**a**) SFI scores in the K3R-treated group were significantly improved on days 6 (*** *p* < 0.001) and 11 (* *p* = 0.032) following sciatic nerve injury relative to the Ctrl group. (**b**) Grip strength was significantly increased in the K3R-treated group on days 4 (*** *p* < 0.001) and 11 (* *p* = 0.012) after injury as compared to the Ctrl group. The improvement in the ipsilateral hind limbs (solid lines) was compared with the contralateral hind limbs (dotted lines). Results are reported as mean ± SEM (*n* = 8 per group).

**Figure 7:**
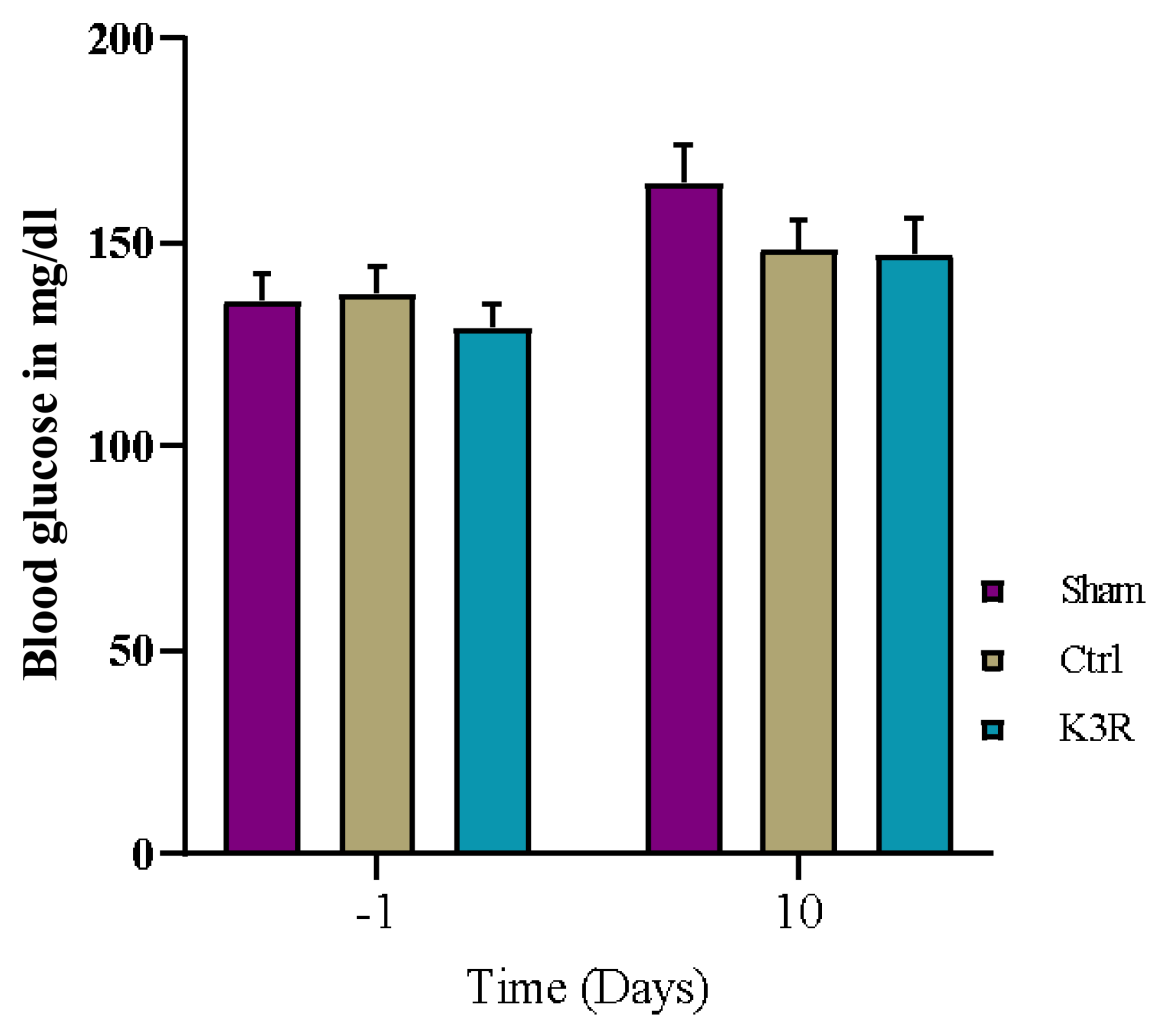
Impact of K3R on glucose level: Two-way ANOVA followed by Tukey’s multiple comparisons test revealed no significant differences in glucose levels between the K3R-treated and the Ctrl groups either 1 day before injury (*p* = 0.708) or 10 days after injury (*p* = 0.995). Data are expressed as mean ± SEM (*n* = 8 per group).

**Figure 8:**
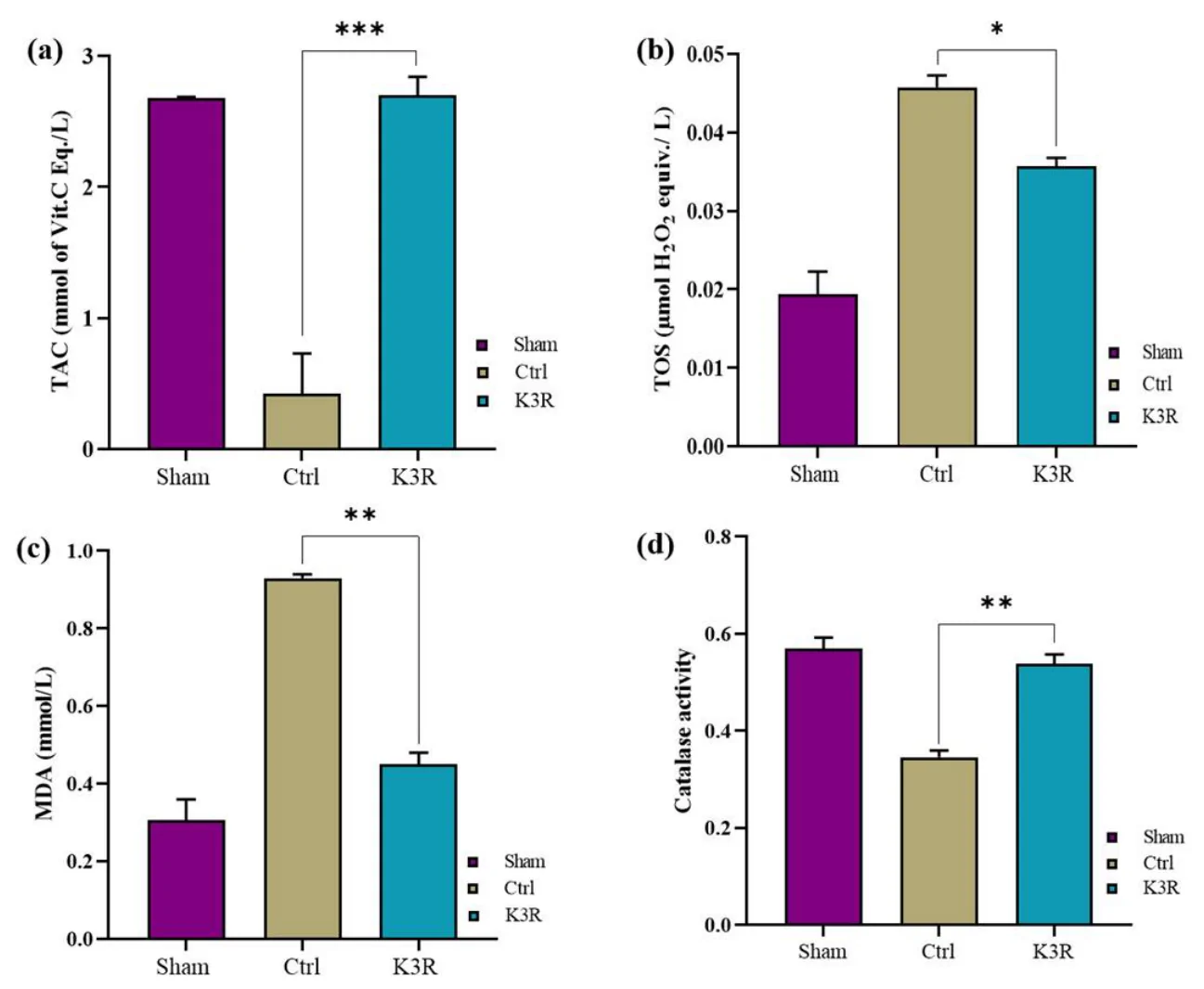
Impact of K3R on oxidative stress markers: Data were analyzed using one-way ANOVA followed by Dunnett’s multiple comparisons test. (**a**) Total Antioxidant Capacity (TAC): TAC levels were significantly higher in the K3R-treated group compared with the Ctrl group (*** *p* < 0.001). (**b**) Total Oxidant Status (TOS): TOS levels were significantly lower in the K3R-treated group (* *p* = 0.021) than in the Ctrl group. (**c**) Malondialdehyde test (MDA): MDA levels were significantly lower in the K3R-treated group than in the Ctrl group (** *p* = 0.004). (**d**) Catalase test: Catalase activity was improved significantly in K3R-treated group compared to the Ctrl group (** *p* = 0.009). Results were represented as mean ± SEM (*n* = 8).

**Figure 9:**
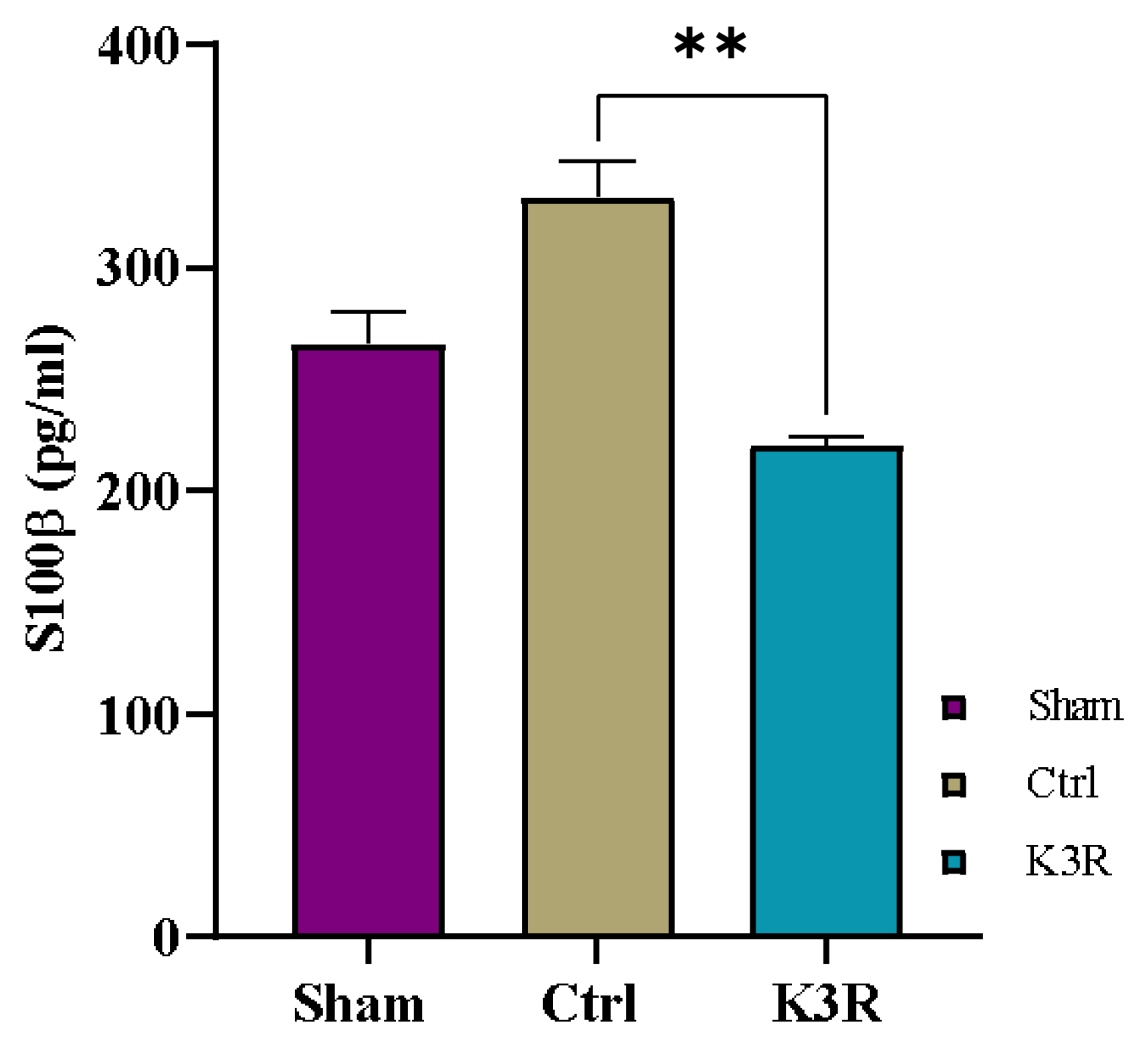
Impact of K3R on S100β protein levels: Serum S100β level was evaluated in the mice. Ordinary one-way ANOVA followed by Dunnett’s multiple comparisons test showed significantly lower S100β levels in the K3R-treated group than in the Ctrl group (** *p* = 0.001). Data were represented as mean ± SEM (*n* = 8).

**Figure 10:**
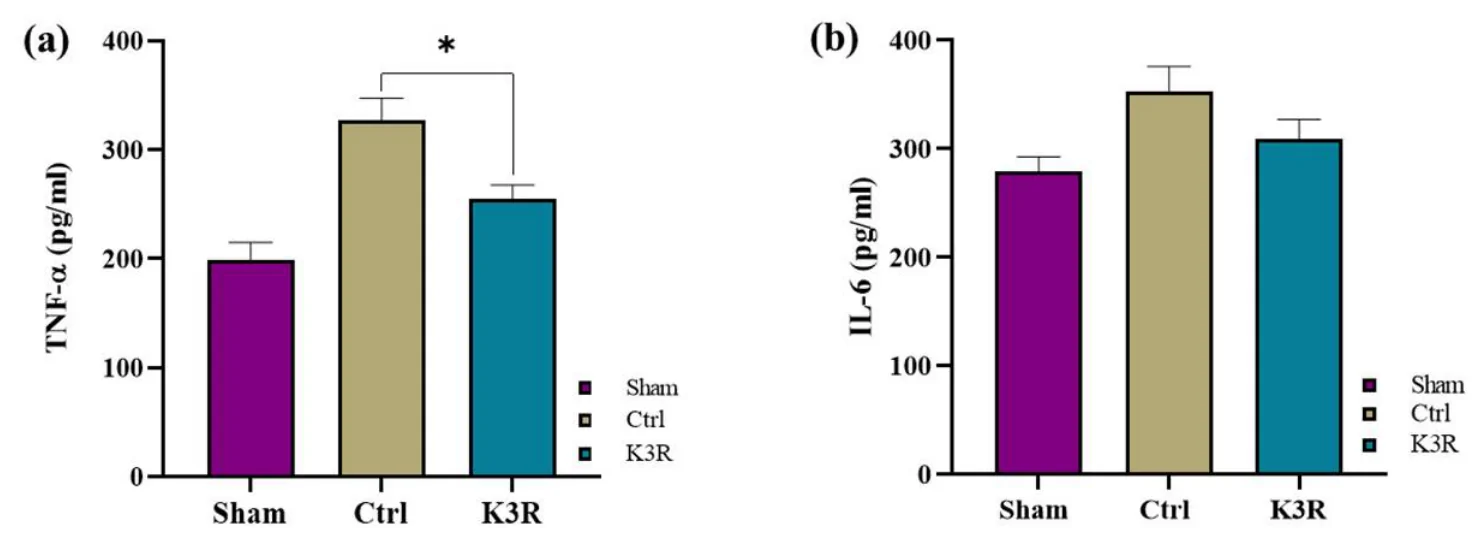
Impact of K3R on TNF-α and IL-6 levels: Serum TNF-α and IL-6 levels were higher in the Ctrl group than in the sham and K3R-treated groups. Data were analyzed using ordinary one-way ANOVA followed by Dunnett’s test for multiple comparisons. (**a**) TNF-α levels were significantly lower in the K3R-treated group compared with the Ctrl group (* *p* = 0.038). (**b**) IL-6 levels showed no significant difference between the K3R-treated and the Ctrl groups (*p* = 0.077). Results were represented as mean ± SEM (*n* = 8).

**Figure 11:**
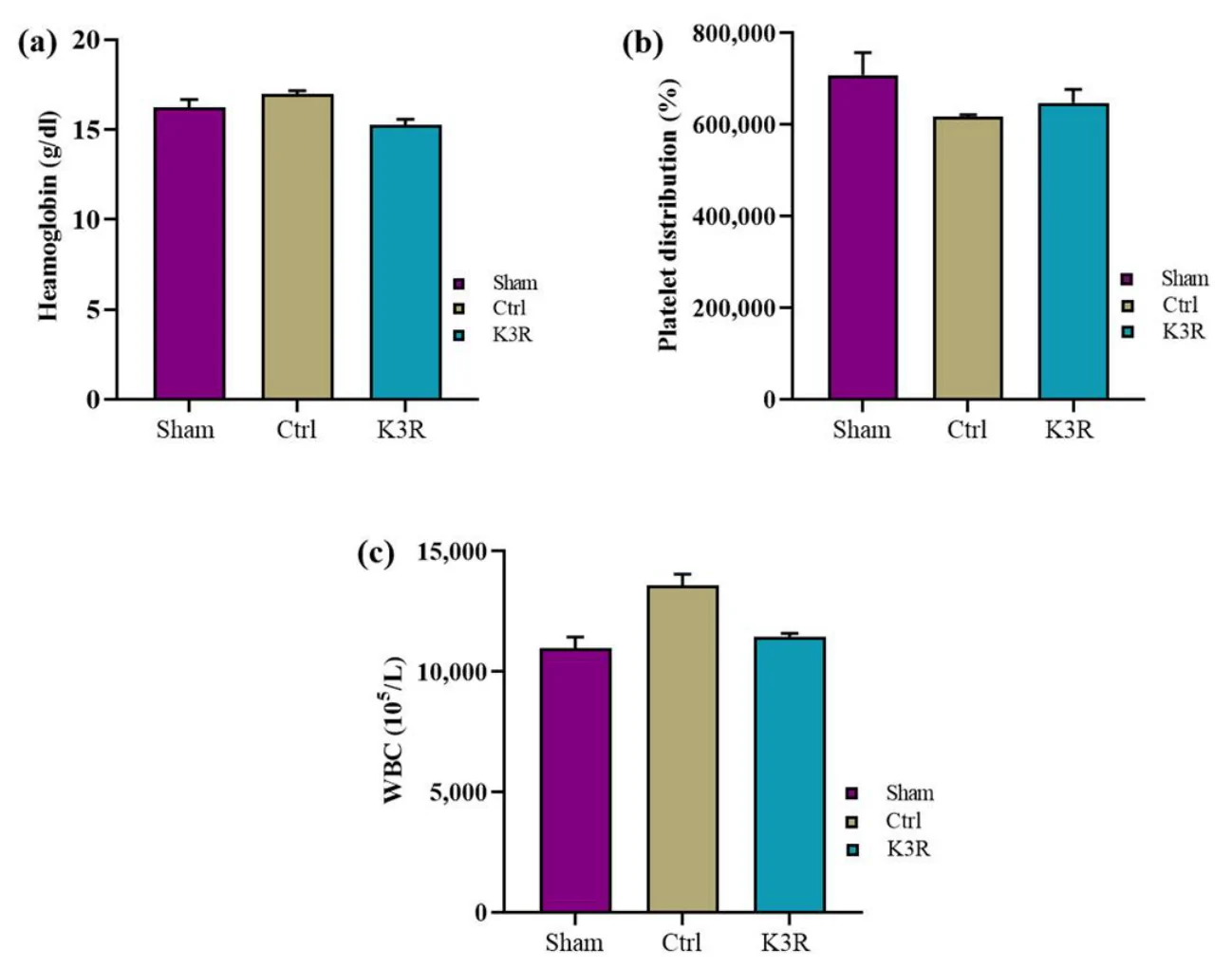
Impact of K3R on hematological indices: Statistical comparisons were performed using ordinary one-way ANOVA, with Dunnett’s test applied for multiple comparisons. (**a**) Hemoglobin levels were significantly lower in the K3R-treated group than in the Ctrl group (*p* = 0.006). (**b**) No significant differences were observed in platelet counts (*p* = 0.805). (**c**) A significant change was observed in WBC counts between the K3R-treated and the Ctrl groups (*p* = 0.46). Values were represented as mean ± SEM (*n* = 8).

**Figure 12:**
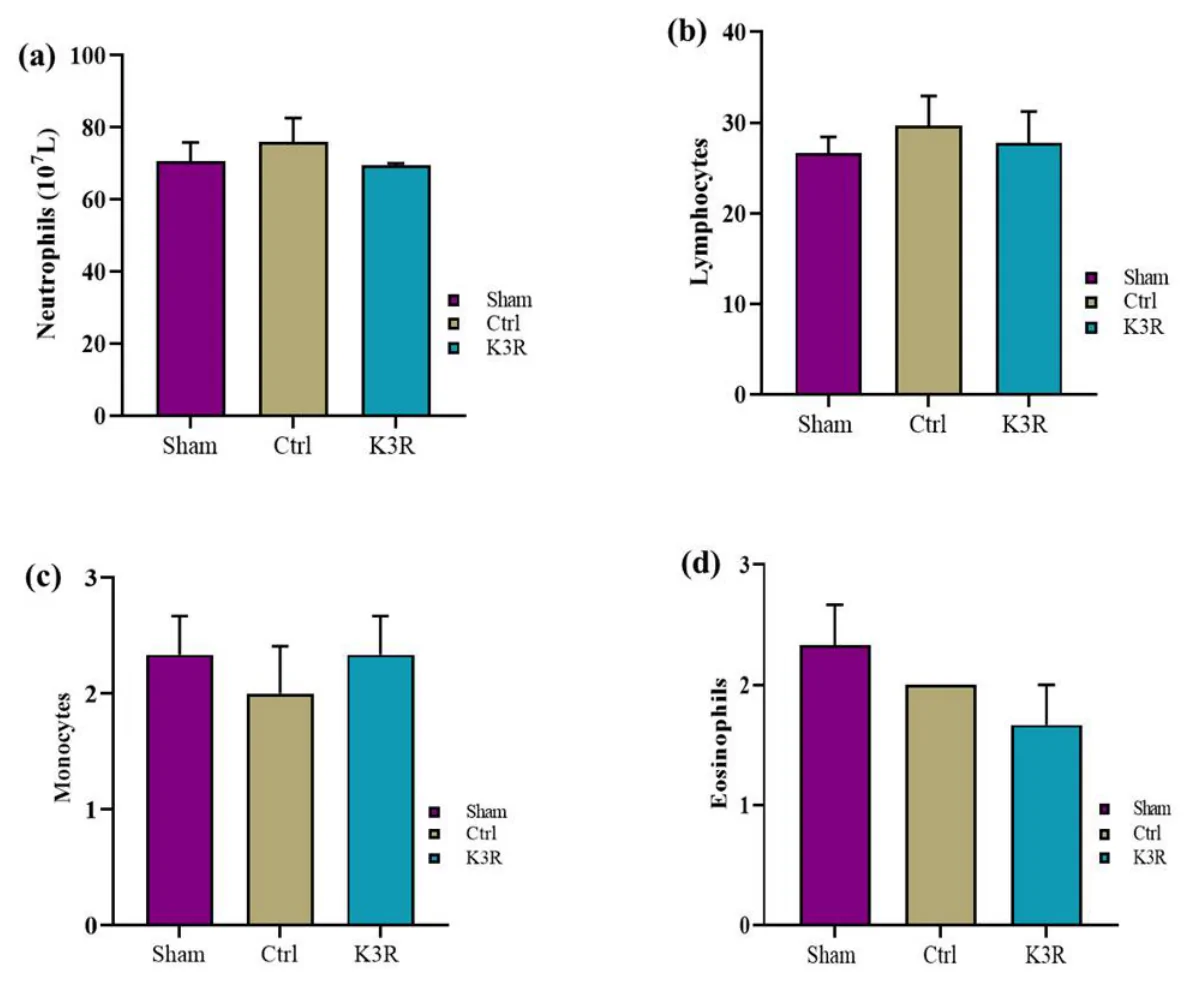
Impact of K3R on differential blood count: Data were analyzed using ordinary one-way ANOVA followed by Dunnett’s multiple comparisons test. No significant differences were observed in (**a**) neutrophil (*p* = 0.687), (**b**) lymphocyte (*p* = 0.869), (**c**) monocyte (*p* = 0.618), or (**d**) eosinophil (*p* = 0.618) counts between the K3R-treated and the Ctrl groups. Data are expressed as mean ± SEM (*n* = 8).

**Figure 13:**
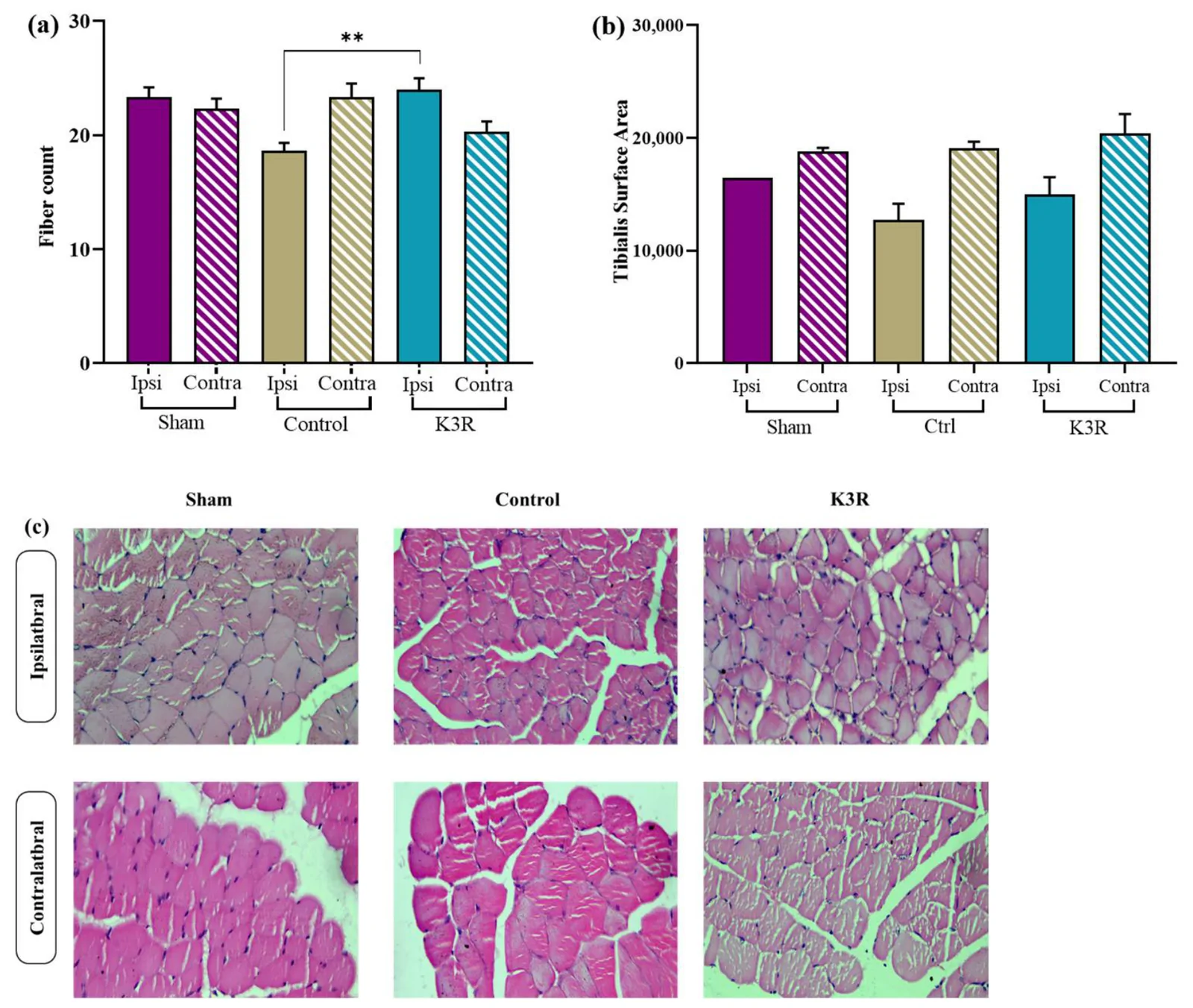
Impact of K3R on the tibialis anterior muscle fiber cross-sectional area: Data were analyzed using ordinary one-way ANOVA followed by Dunnett’s multiple comparisons test. (**a**) Fiber counts were determined from contralateral and ipsilateral sections of the tibialis anterior muscle. The K3R-treated group showed a significantly higher fiber count compared to the Ctrl group (** *p* = 0.007). (**b**) The surface area of muscle fibers was measured in contralateral and ipsilateral sections of the tibialis muscle. No significant difference was observed between the K3R-treated and the Ctrl groups (*p* = 0.822). (**c**) Under a compound microscope, a cross-section of the tibialis anterior muscle was observed; images were captured at 40× magnification using H&E stain. Data are represented as mean ± SEM (*n* = 8).

## Data Availability

The original contributions presented in this study are included in the article. Further inquiries can be directed to the corresponding author.

## Abbreviations

The following abbreviations are used in this manuscript:

Ctrl	Control
K3R	Kaempferol-3-O-rutinoside
PNI	Peripheral nerve injury
HPL	Hot plate latency
WRL	Withdrawal reflex latency
SFI	Sciatic functional index
TAC	Total antioxidant capacity
TOS	Total oxidant status
MDA	Malondialdehyde
DMEM	Dulbecco’s modified Eagle medium
DMSO	Dimethyl sulfoxide
PL	Print length
TS	Toe spread
IT	Intermediate toe spread
